# Supplementary figures and images for: IFN-γ Critically Enables the Intratumoural Infiltration of CXCR3+ CD8+ T Cells to Drive Squamous Cell Carcinoma Regression
Source: Cancers (Basel). 2021 Apr 28;13(9):2131. doi: 10.3390/cancers13092131 (PMC8124943; doi:10.3390/cancers13092131)

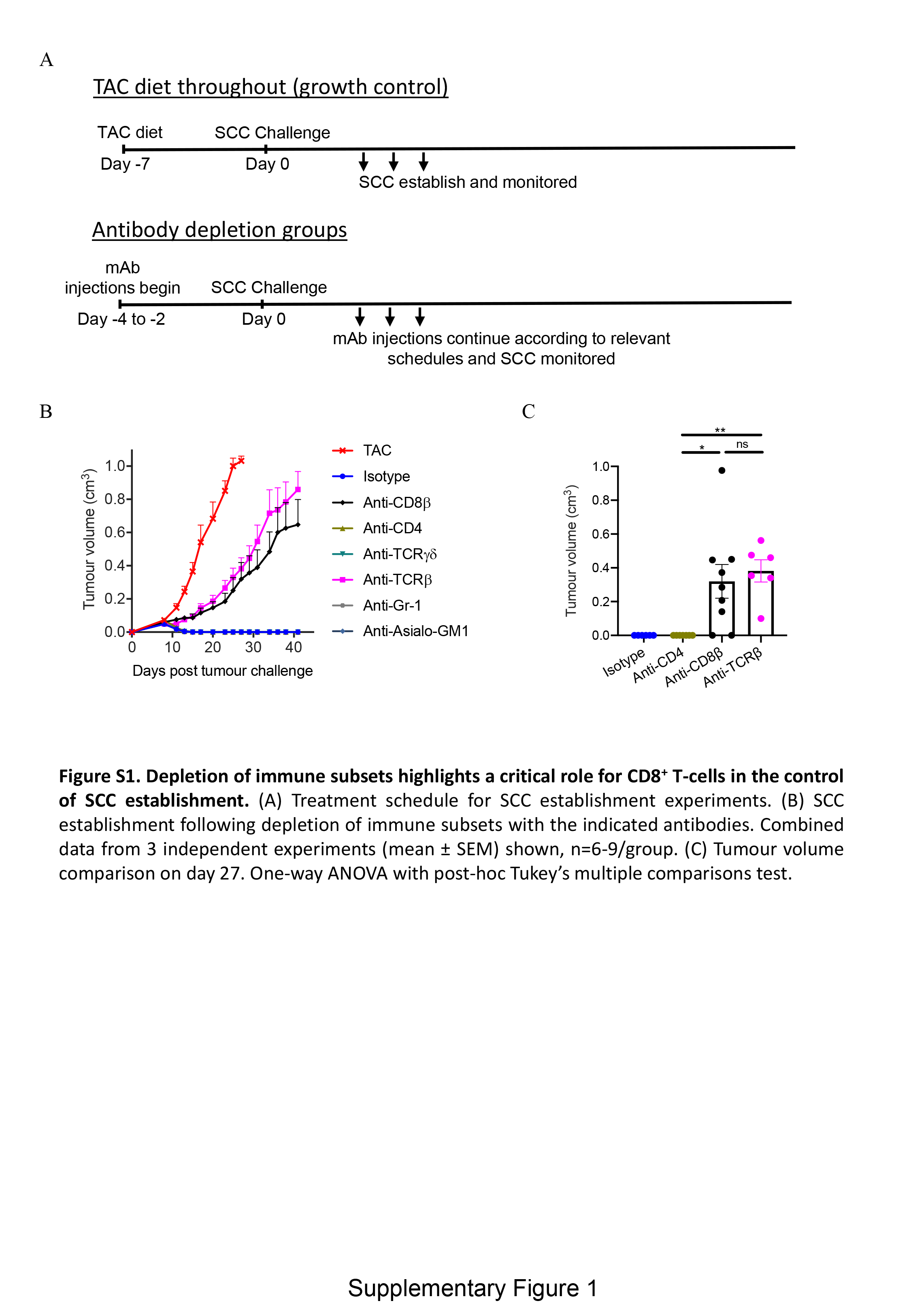

Supplement: Supplementary file 1 [file cancers-13-02131-s001.zip › Supplementary Figure 1.tif]

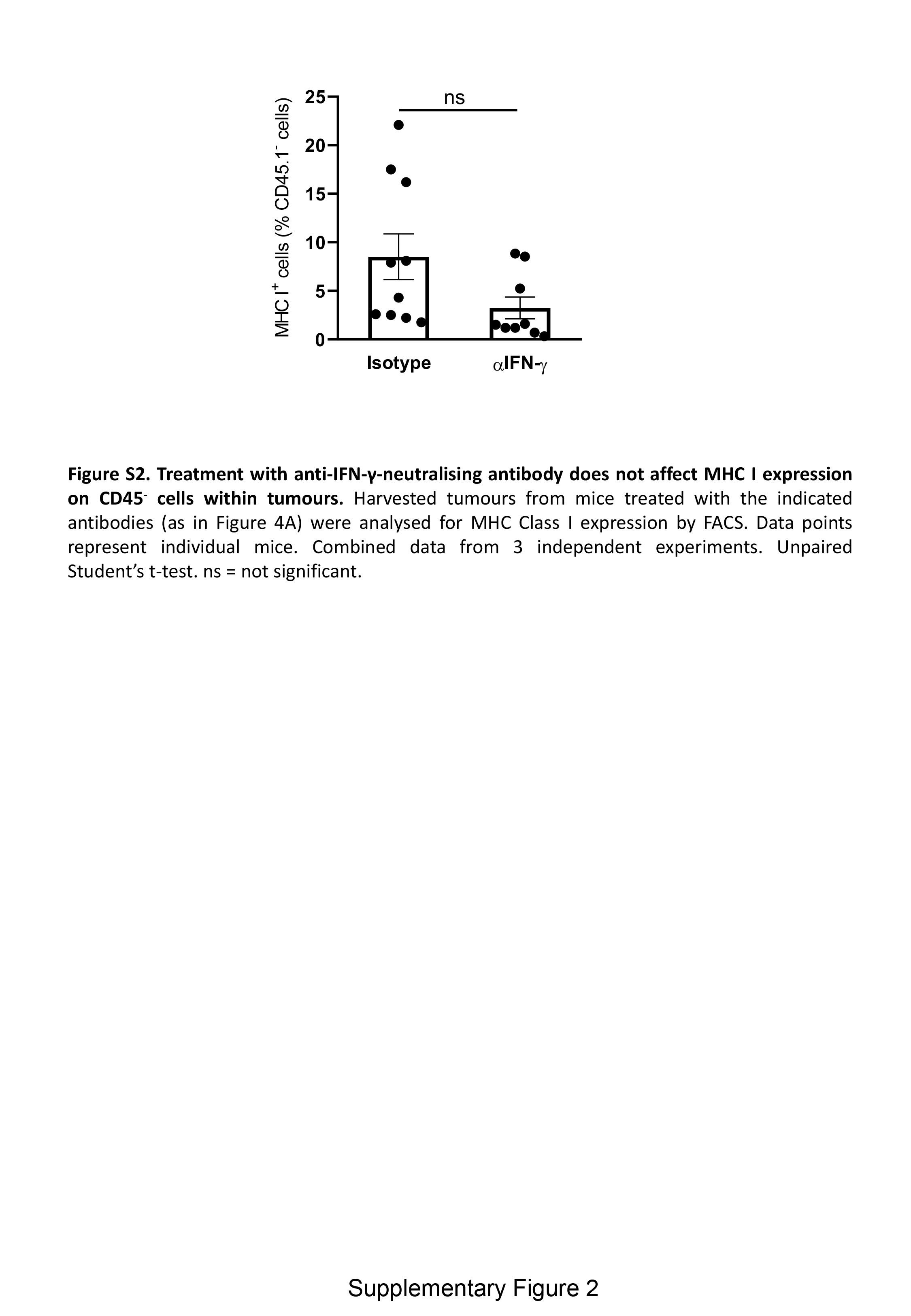

Supplement: Supplementary file 1 [file cancers-13-02131-s001.zip › Supplementary Figure 2.tif]

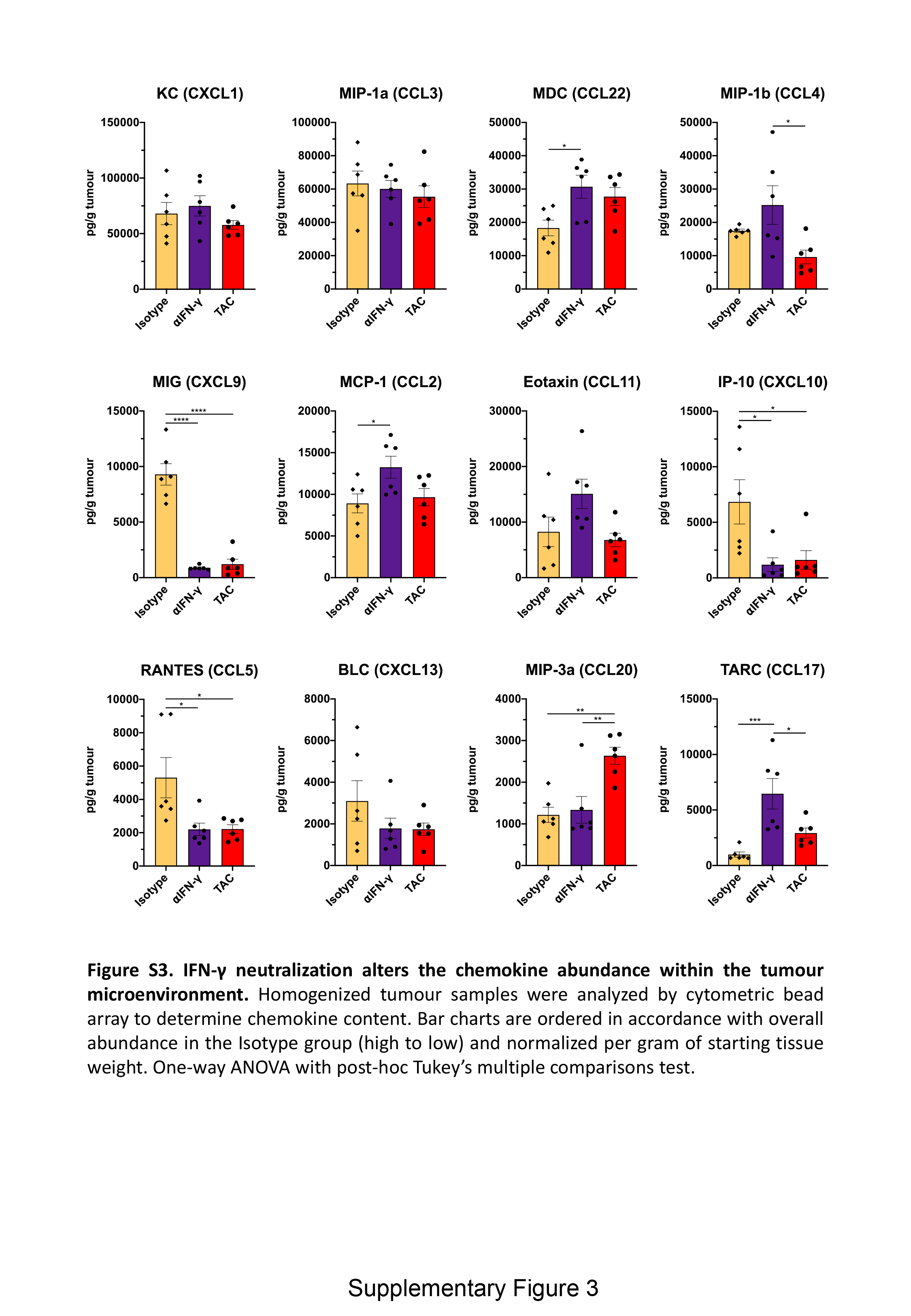

Supplement: Supplementary file 1 [file cancers-13-02131-s001.zip › Supplementary Figure 3.tif]

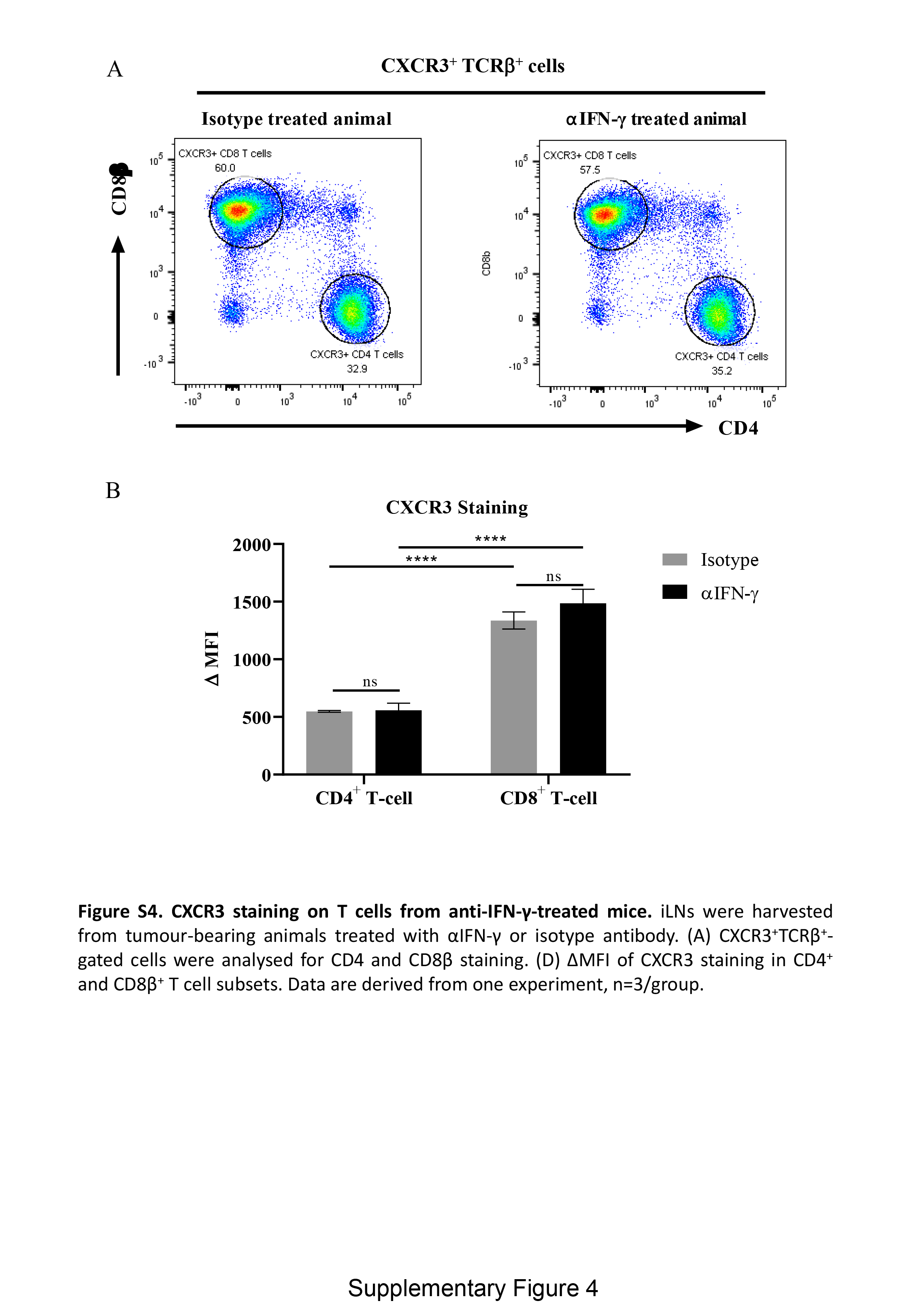

Supplement: Supplementary file 1 [file cancers-13-02131-s001.zip › Supplementary Figure 4.tif]

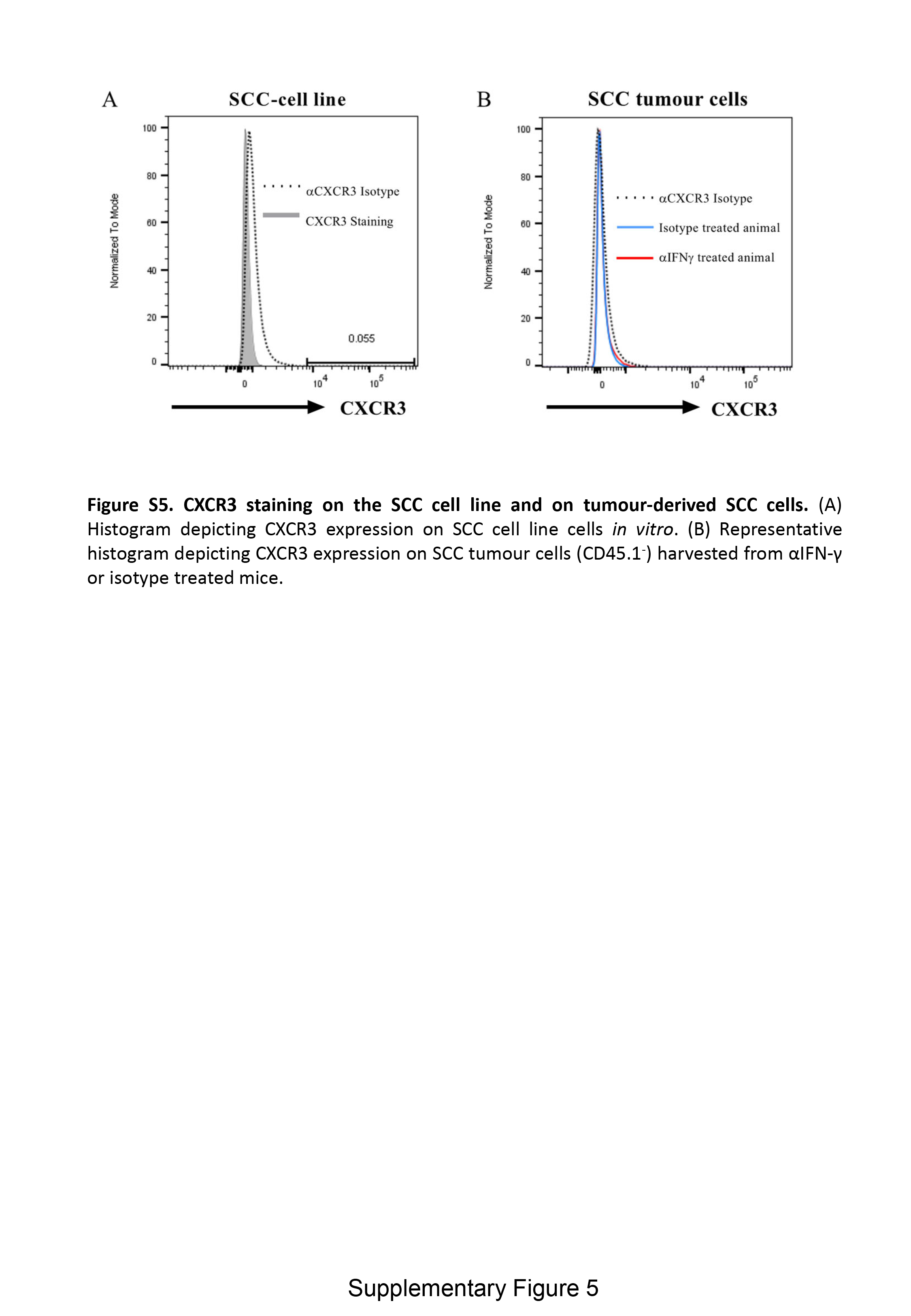

Supplement: Supplementary file 1 [file cancers-13-02131-s001.zip › Supplementary Figure 5.tif]

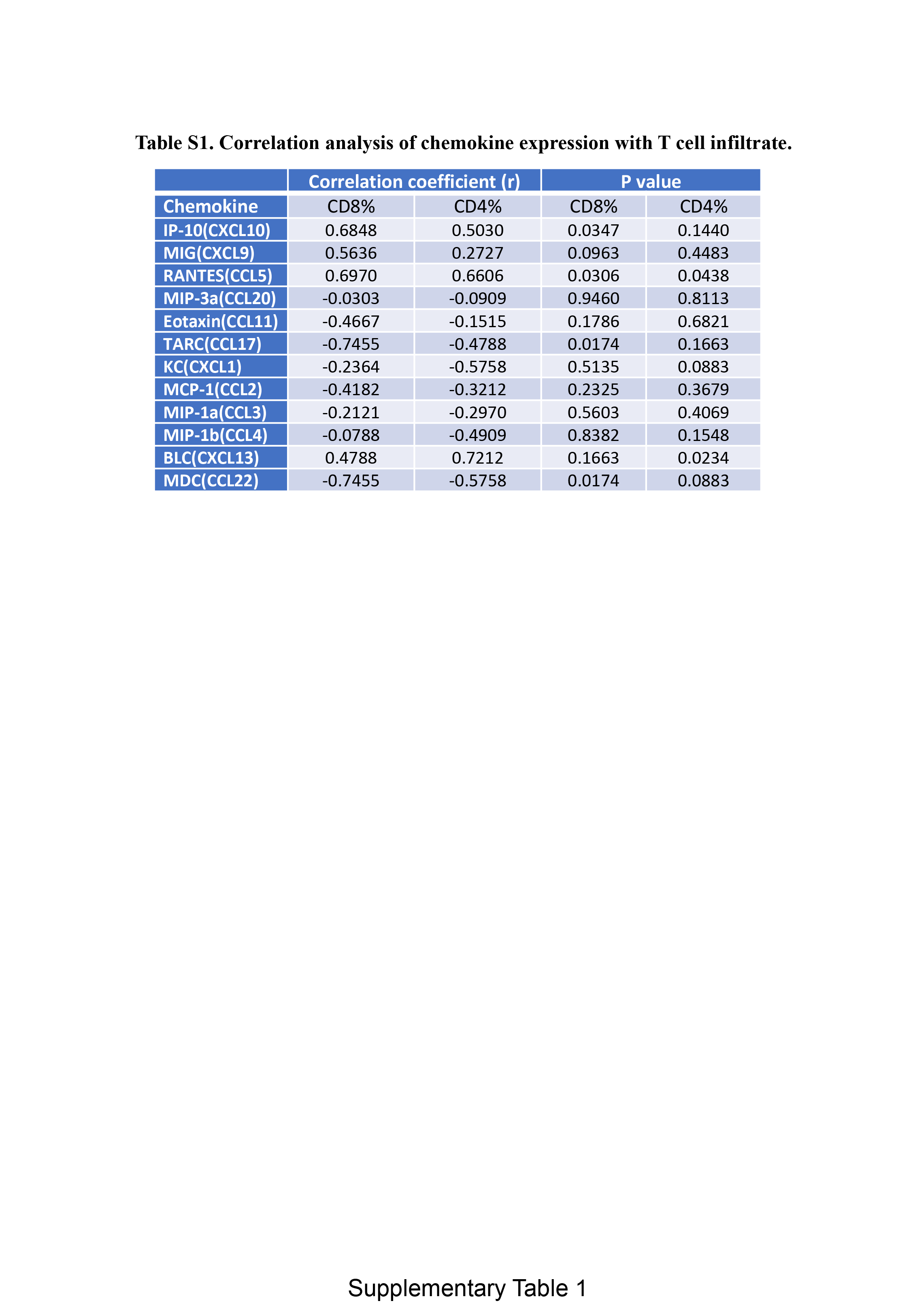

Supplement: Supplementary file 1 [file cancers-13-02131-s001.zip › Supplementary Table 1.tif]

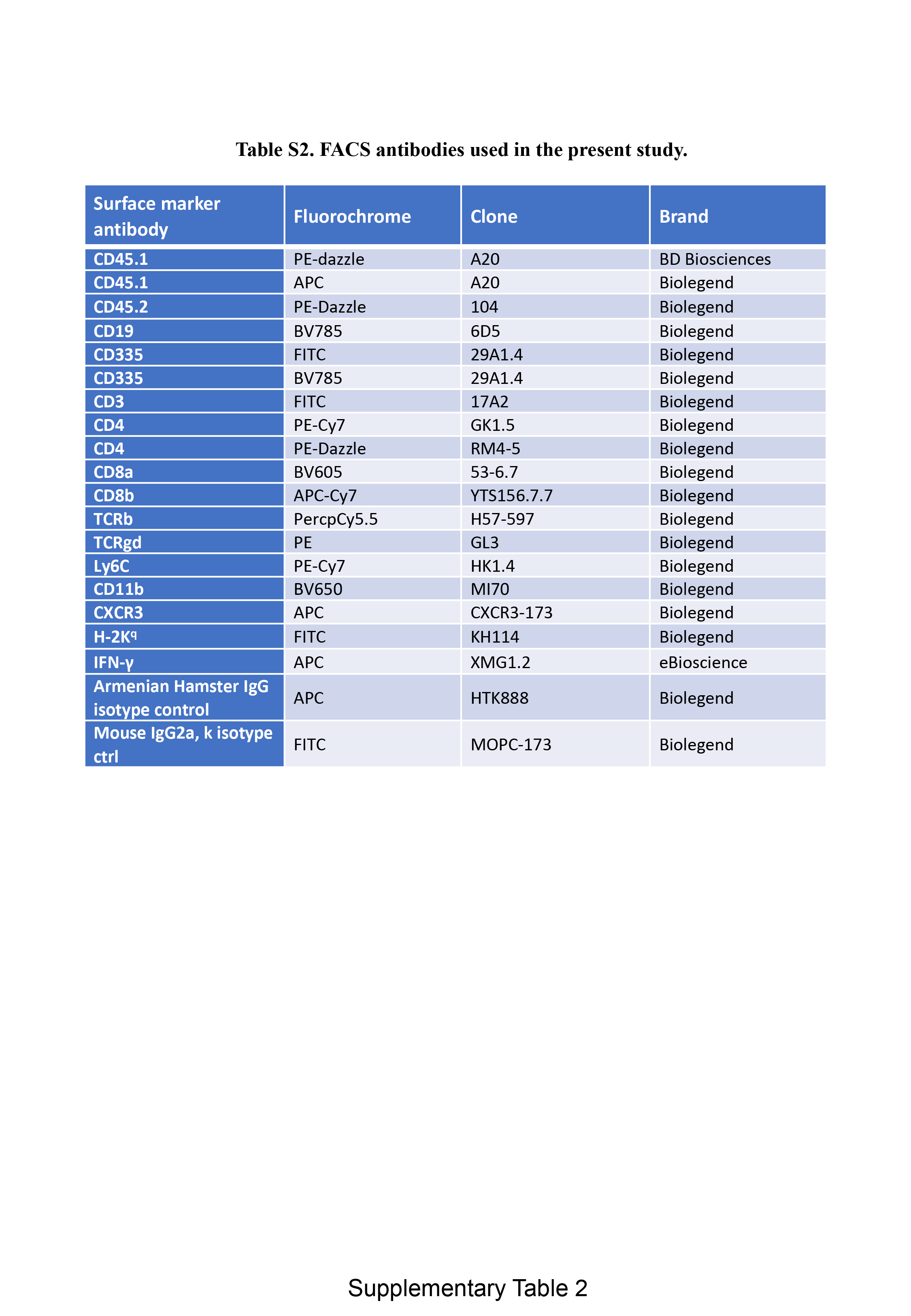

Supplement: Supplementary file 1 [file cancers-13-02131-s001.zip › Supplementary Table 2.tif]
